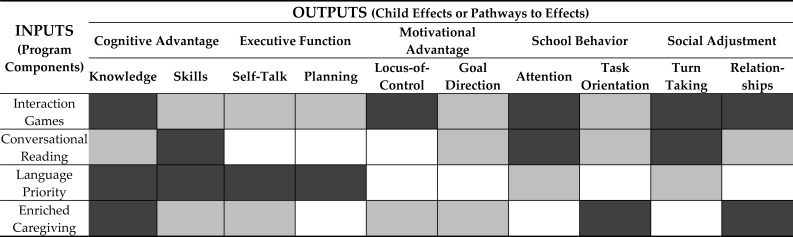# Mental Health and Social Development Effects of the Abecedarian Approach

**DOI:** 10.3390/ijerph18136997

**Published:** 2021-06-30

**Authors:** Joseph Sparling, Sharon Landesman Ramey, Craig T. Ramey

**Affiliations:** 1 Frank Porter Graham Child Development Institute, University of North Carolina at Chapel Hill, Chapel Hill, NC 27599, USA; 2 Melbourne Graduate School of Education, University of Melbourne, Parkville, VIC 3010, Australia; 3 Fralin Biomedical Research Institute, and Departments of Psychology, Neuroscience, and Human Development, Virginia Tech, Roanoke, VA 24016, USA; slramey@vt.edu (S.L.R.); ctramey@vt.edu (C.T.R.)

**Keywords:** Abecedarian, mental health, social development, language development, equity

## Abstract

The Abecedarian Approach is an early intervention and contains a broad-spectrum adult/child curriculum. The Approach has been studied in three longitudinal randomized controlled trials in the USA, starting in 1972 and continuing today. Recent research studies in multiple countries have examined the Abecedarian Approach during the first three years of life. The collective findings from these studies lead to the conclusion that human development is malleable, especially in the years before school entry, and that high-quality early intervention exerts positive, early, and long-lasting influences on human development, including social development and mental health.

## 1. Introduction

A broad-spectrum program such as the Abecedarian Approach attempts to influence multiple strands of development in a holistic way. “Broad-spectrum curricula exist because professionals who facilitate development recognize that each child is more than a collection of skills and attitudes. The ‘more’ can be seen in the pattern by which the skills fit together and reinforce each other. The broad-spectrum curriculum is congruent with the goal of developing an individual who is internally integrated and whose skills are generalized across many situations of life. Because of its emphasis on life situations, a young child’s broad-spectrum curriculum, when being implemented, looks like play. This type of curriculum comprehensively spans large blocks of time and is incorporated into all or most of the things the child does during the day” [1] (p. 2).

The Abecedarian Approach is distinctive in its design and research and in its persistent and pervasive focus on adult-child interaction. It is the only educational program that begins at the child’s birth, continues until the child enters school, is supported by over 40 years of high-quality scientific research [2], and has been effectively implemented through multiple methods of delivery (child care, home visits, and play groups). The Approach was first studied in three longitudinal randomized controlled trials in the USA: the Abecedarian Project [3], Project CARE [4], and the Infant Health and Development Program [5].

The Abecedarian Approach has been implemented in multiple countries. The Approach has been adapted and used as a birth to age three or birth to age five program in Australia, Canada, China, Denmark, France, India, Jordan, Mexico, Mongolia, Pakistan, Singapore, and Zambia [6,7,8,9,10,11]. Some of these uses were research studies while others were training and implementation applications. Each of these efforts provides new insights and creativity in making the Abecedarian Approach a useful and widely used resource.

Abecedarian studies that were conducted after the first study, were not traditional replications in that they did not simply repeat the original study at another time or place. Each study tried to produce new knowledge by varying some aspect such as the method of program delivery, setting, length of treatment, or program staffing. These are components that have been varied across the Abecedarian research studies.

- Samples: children from low-resource families, children born prematurely and at low birth weight, children with cerebral palsy, children from various countries, Indigenous children, children in an orphanage.
- Method of program delivery: center-based group care, family child care homes, play groups, home visiting.
- Length of intervention: birth to 36 months of age, birth to school entry.

In the early 1970s, the original Abecedarian researchers asked the question: Could a positive early childhood experience really help historically low-achieving children develop in a pattern similar to the general population of children and have greater success in school? To begin the process of answering this question, the Abecedarian Approach was created to serve as the core of the “educational treatment” in the Abecedarian Project, a rigorous longitudinal research study. The investigators hypothesized that providing theory-based, active, contingent experiences to children across the very early years of development could significantly improve their academic achievement and social adjustment once they reached school. Full day child care was chosen as the platform for delivering these experiences in the first study because it allowed the researchers the opportunity to verify that the educational program was being delivered faithfully and in sufficient amounts.

The theoretical underpinnings of the Abecedarian Approach were derived from three classic sources. Vygotsky’s [12] concept of adult-mediated activities is the basis for the emphasis that Abecedarian places on the adult’s role in facilitating and adding emotional and education value to caregiving, play, and games. Scaife and Bruner’s [13] explanation of joint attention is the basis for the Abecedarian focus on graded prompts in early book reading and play. Piaget’s [14] sequencing of developmental progression provides specific behavioral goals for the Abecedarian activities, especially the Interaction Games. These concepts are embodied in the Abecedarian Approach through four core educational program elements.

## 2. What Is the Abecedarian Approach?

A key Abecedarian feature is that its educational program begins at birth [15] and supports this pivotal early period of learning and brain development. This dramatically separates it from other well-known early childhood programs that begin at age 3 or 4 years. At the time these other programs enroll children, the Abecedarian Approach has already had a significant part of its effect.

The adult-child curriculum of the Abecedarian Approach is a strong set of teaching and learning strategies implemented through intentional adult-child interactions that are explicitly described and illustrated in the Abecedarian program resources, activities, learning strategies, and training.

In creating this Approach for the Abecedarian Project in the early 1970s, we strove to capture the emerging scientific knowledge about how infants and young children grow and develop and to translate this technical information into playful activities and educational interactions. We attempted to create activities that could be understood and used by parents and by all adults working with and caring for young children. The Approach incorporated many features of a stimulating home environment as well as some features of a high-quality child care center.

These are the four elements of the Abecedarian Approach briefly described:

- Language Priority—making each part of the child’s day an opportunity for talking, listening, responding, and taking turns. Young babies’ glances and gestures are accepted as important parts of a two-way conversation.
- Conversational Reading—reading books interactively, emphasizing the child’s active role. The adult provides graded or hierarchical prompts to gradually elicit more developmentally advanced responses from the child. The word “conversational” emphasizes the back-and-forth, reciprocal nature of this type of reading.
- Interaction Games—playing interactively through adult-child games tailored to the child’s interests and developmental level. The games appear easy on the surface but challenge the adult to find just the right level and variation for the individual child. While the action of the game is simple, the significance to the child’s development can be profound.
- Enriched Caregiving—incorporating educational content and social-emotional connection into the child’s daily care routines such as feeding/eating, changing diapers/toileting, bathing/washing hands, getting dressed/undressed. Adults enhance the basic level of care they provide by emphasizing its social-emotional aspect as well as incorporating explicit educational content such as shapes, sizes, colors, numbers, and processes.

Each element is one way of looking at adult-child interactions. In the Abecedarian Approach, interactions occur intentionally, individually, and frequently. If the adults are educators, they have written plans. If they are parents, the “plans” may be more informal. This allows the adults to engage in the activities intentionally and focusing on individual children. Most of the interactions are performed one-on-one (or one adult to two children when the children are developmentally ready) allowing the adult to tailor content and responses to the child’s individual needs. Each of the elements of the Abecedarian Approach is incorporated into the child’s day multiple times, the repetition providing many opportunities for practice.

Since the Abecedarian Approach focuses on multiple strands of child development, one way to think about the Approach is to consider how the parts of the Abecedarian adult-child curriculum are intended to relate to the observed or hypothesized Abecedarian effects or outputs. Table 1 provides a summary this set of relationships in an Input-Output Map.

The map presents a rudimentary topographical view of the intersection between the Abecedarian Approach (the program inputs) and the Abecedarian child effects (the program outputs). The darker areas are hypothesized to be areas of strong influence, the lighter areas to be areas of moderate influence, and the unshaded areas to represent non-hypothesized or minimal influence.

Some child care providers who read about the Abecedarian Approach notice that some of the ideas in the four elements of the Approach seem familiar and are similar to some of their current practices. Their response is, “This is not special, I already do it” [16]. It is true that many of the Abecedarian practices may be seen in good child care programs. Yet, within most programs the practices are not used with enough individualization, frequency, and intentionality to produce the profound effects that are shown in the Abecedarian research. “The innovation of the Abecedarian Approach was in bringing together all the elements of high quality and holding them steady over an extended period of time at a level of consistency that would yield significant, measurable, long-term benefits for vulnerable children and families” [17] (p. 6).

The research reported below does not imply that students from families of low economic and low educational backgrounds who do not participate in a program such as the Abecedarian Approach tend to have immutable developmental profiles that are not amenable to change by the school after age five. The research demonstrates that, on average, children from low-resource backgrounds who have experienced an early Abecedarian program enter school with a developmental and educational advantage compared to children from similar backgrounds who have not experienced the program and that the advantage accrues to their benefit throughout their school years and into middle adulthood.

## 3. Mental Health and Social Development Effects from Abecedarian Interventions

Since the Abecedarian Approach has been studied for a number of decades, there have been hundreds of peer-reviewed journal articles and book chapters published on the research findings of this program. Multiple Abecedarian studies show that intervening broadly for children from low resource families yields a variety of early and long-lasting positive outcomes [2]. Often, the Abecedarian Approach has been viewed as a cognitive intervention, and indeed, it does have strong and lasting cognitive [18] and academic achievement [19] effects. However, that view masks the breadth of the Abecedarian intervention and the breadth of its effects. In this section we focus on research findings that reveal the other Abecedarian effects—especially those that are traditionally labeled as social development and mental health. The studies discussed below were chosen because they reach from the first year of life through the fourth decade of life and represent findings on various aspects of social development and mental health across that span.

### 3.1. Attachment

Because the first Abecedarian study, called the Abecedarian Project, was delivered through group child care, the researchers wanted to know if there was a positive or negative effect from the child care experience itself. At one year of age the infants and their mothers were observed in the Ainsworth Strange Situation paradigm [20]. This observational study, early in the history of the Abecedarian Project, showed that Abecedarian child care was not associated with increased insecure attachment and did not negatively change, and sometimes enhanced, the associations between the infant-mother attachment and the mother’s involvement and warmth toward the infant during the first year of life [21]. On another occasion the children who attended Abecedarian child care were observed in a situation designed to heighten attachment behaviors; both their mothers and a favorite infant-day-care teacher were present. Children overwhelmingly stayed near and interacted with their mothers rather than their teachers, indicating that the attachment bond to the mother had indeed been formed. Additionally, they chose their mothers as the help giver when faced with a mildly difficult problem [22].

### 3.2. Communicative Initiations

In the first Abecedarian study video data were collected on how 20-month-old infants from low resource families use intentional communication in a free-play setting. Children who received the Abecedarian program were compared to randomly assigned children who received other programs or stayed at home. They were also compared to a group of normally developing middle-class infants. The frequency of communicative initiations and the proportion of spontaneous communicative initiations—classified as “showing” and “requesting”—were used for the comparison. Receiving the Abecedarian Approach through child care significantly enhanced both the frequency and the developmental level of the communicative initiations of infants from low-resource families [23].

### 3.3. Language Development

A 2019 impact study sought to determine if the classic Abecedarian Approach, in the current time and in an on-going service setting, could improve the early developmental progress of historically underachieving Canadian children, including those of First Nations (FN) status. A treatment group received the Abecedarian Approach in a child care center supplemented by home visiting, and a control group received child care at other centers or care at home. Children were assessed on the language scale of a standardized instrument, the Brigance Screen [24] at enrollment and yearly thereafter. There were 80 children in the study ranging from two months to five years in age. Participation in the treatment program led to average scores on the language assessments. Although both genders in the treatment group experienced net increases over the control group in language development, the girls did better than the boys. Additionally, it is of particular interest that in the treatment group, the FN children began at a lower language level than the non-FN children, but both groups progressed at about the same rate. In contrast, under the control condition the non-FN children made slow progress and the FN children gained almost no ground. The gap between the FN treatment group and the non-FN control group at baseline was completely closed by year two of the study—at which point these two groups had identical scores [7].

### 3.4. Locus of Control

An academic locus-of-control measure [25], collected during the kindergarten and first grade years on the first two cohorts of the Abecedarian Project, showed that the untreated children had lower perceptions of control over their academic success than did their middle and upper socio-economic classmates. However, for the Abecedarian treatment children, beliefs in personal control over successful academic performance increased to approximately the same level as that of their low-risk classmates. For the treatment children in the study, academic locus-of-control was positively related to task-orientation, less distractibility, and internal motivation [26]—providing a possible explanation for how beliefs (locus-of-control) may be translated into positive classroom behaviors. Elementary school children who previously experienced the Abecedarian Approach may have a greater sense of internal control over their own academic performance.

### 3.5. Problem Behavior

A randomized clinical trial in eight states, the Infant Health and Development Program (IHDP), examined the Abecedarian Approach intervention with children born preterm and at low birth weight. The parents completed the Achenbach Problem Behavior Checklist [27] in each of their child’s first three years of life. At 24 and 36 months of age, behavior problem scores for the IHDP treatment group were significantly lower than for the control group (Ramey et al., 1992). The Abecedarian intervention had a greater cognitive effect for children with higher initial behavior problem scores [28].

### 3.6. Cortisol

Cortisol is a hormone that has been used to study human responses to stress. During and following an Abecedarian intervention in the first three years of life in a Romanian Orphanage, researchers gathered saliva cortisol samples from treatment, control, and general population comparison children. Saliva samples were collected at 8 AM, noon, and 7 PM on two consecutive days at age two and again at age three. Saliva samples were also collected before, during, and after a stressful event. The cortisol levels indicated that children who had experienced the Abecedarian Approach were better at handling the general stress of the day and specific stressful events than were the control group. In addition, for the untreated group there were some negative correlations between cortisol levels and cognitive development [29,30].

### 3.7. Risky Behaviors

Subjects in the Abecedarian low birthweight study (IHDP) received a follow-up assessment at 18 years of age using questions from the Youth Risk Behavior Surveillance System [31]. Items from this survey related to conduct problems, suicidal ideation/attempts, smoking, alcohol and marijuana use, and risky sexual activity. Youth who had received the Abecedarian Approach in the first three years of life had fewer risky behaviors at age 18 than the randomly assigned control group [32].

### 3.8. Depression

Young adults who were in the treatment and control groups of the Abecedarian Project completed the Brief Symptoms Inventory [33] at age 21. The group that received the Abecedarian early childhood treatment reported fewer symptoms of depression. The protective effects of the early childhood program were further supported by a significant home environment by treatment interaction. There were no significant mean differences in the home environments of the treatment and control groups, but the negative effects of lower quality home environments on young adult depressive symptoms were almost entirely offset by the Abecedarian treatment [34].

### 3.9. Healthy Life Style

Abecedarian young adults showed a significant treatment-related reduction in reports of recently using marijuana. Use of other drugs and alcohol were not different between the groups. There was a trend in the Abecedarian follow-up data for fewer individuals who had early childhood intervention to report being regular smokers in young adulthood compared with controls. Additionally, for participants who received the Abecedarian treatment, the odds of reporting a healthier, more active lifestyle in young adulthood were 3.92 times greater compared to participants from the control groups [18].

### 3.10. Criminal Behavior

A long-term follow-up study summarizing data gathered at ages 21, 30, 34 looked at the crime-reducing impacts of the Abecedarian early childhood program. Proportionately, more women than men who participated in the early childhood Abecedarian program had less criminal activity than the control group. This gender difference occurred because the home environments were worse for the girls, with corresponding greater scope for improvement by the program. For both genders, treatment effects were larger for the least advantaged children, as measured by their mother’s education at the beginning of the early childhood intervention. The dollar value of the social cost of criminal activity averted was higher for men because they committed more costly violent crimes [35].

### 3.11. Social Services Benefits

The percentage of the Abecedarian treated and control groups at age 30 who worked full time for at least 2/3 of the preceding 24 months showed that the odds of being employed for those in the Abecedarian treated group were more than twice the odds for those in the control group. Similarly, the group with Abecedarian early childhood treatment had slightly higher job prestige scores than the control group but the difference did not reach statistical significance. Approximately a quarter of each group had married by age 30, but the odds of being the head of one’s own household were almost twice as high for the treated group. Administrative data on use of government welfare funds showed that, within an 89-month time window, individuals in the control group were 6 times more likely to receive benefits at least 10% of the time [36].

### 3.12. Hypertension and Other Health Indicators

The Abecedarian investigators recruited all study participants for an adult physical examination whose findings could be compared as a function of early childhood treatment. At the time of the medical study, participant age ranged from 32 to 39 years and were an average of 35.4 years. Findings for the study indicated that males who had received Abecedarian treatment had significantly lower blood pressure than did males in the control group. None of the males in the treatment group exhibited the complex of conditions called metabolic syndrome (large waist, unfavorable cholesterol levels, and elevated blood pressure) known to be predictive of heart disease, whereas 25% of the males in the preschool control condition did so. Females did not show the same treatment effects as males, but females who had been in the early childhood Abecedarian treatment group were less likely to be affected by abdominal obesity and were less likely to fall in the pre-hypertension category than were the control females [37].

### 3.13. Social Decision-Making

When the Abecedarian treatment and control alumni were in their early 40s, they and a sample of general population young adults played the “ultimatum game.” In this game they were asked to accept or reject offers that divided an amount of money between themselves and another (unseen) player. Social decision making was measured—especially in terms of how the subjects would choose to divide resources. The Abecedarian treatment alumni opted for greater fairness and equity in the division of resources than did the control group or a general population sample [38]. *Education Week* [39] summarized these findings as “Preschool lessons in fair play may last a long time”.

### 3.14. Does Participation in the Program and the Curriculum Really Matter?

The year-by-year levels of participation of individual children and families showed a clear association between participation levels and cognitive progress at ages two and three [40]. For each year from ages 1–3, the days attended by the child in an Abecedarian center, the number of home visits, and the number of parent meetings attended predicted cognitive advances above and beyond the child’s background characteristics such as maternal education and very low birth weight.

Knowing that the level of family and child participation in the low birthweight study made an impact, the original researchers looked further into program process variables. They especially used the data related to the learning activities that comprise the four elements of the Abecedarian Approach. They analyzed these curriculum-related records to see if for individual children a basic measure of curriculum such as a count of Abecedarian learning activities per child-care day and per home visit could predict child cognitive outcome. First, the researchers statistically equalized the effects of initial status variables (such as maternal age, maternal education, and child neonatal health status) and the effects of family and child participation. After all those effects were accounted for, the analysis showed that the rate of delivery of Abecedarian learning activities still added significantly to the prediction of child 36-month Stanford Binet IQ [41]. So, not only do families and children need to participate in the program, but they need to get sufficient amounts of the learning activities or curriculum while they are there.

Another group of researchers continued to look into this topic. They created an Active Experience Index comprised of the level of interest parents showed regarding the Abecedarian learning activities during home visits plus the degree of mastery of the learning activities that children achieved in the child care center. After controlling for child and family initial status variables, they showed that Active Experience improved the prediction of child 36-month IQ to even a greater degree than did the simple rate of curriculum delivery [42]. This analysis shows that it is important to involve both family and child and particularly to capture the interest of the family and assure the child’s mastery of educational activities.

### 3.15. Cognitive Advantage Plus Social Adjustment Plus Motivational Advantage

What early factors bear on later achievement in school and life? Following a mediation of effects strategy, an analysis of several early interventions showed that a predictive model comprised of cognitive advantage, plus social adjustment, plus motivational advantage accounted for 100% of the preschool effect on years of education at age 21 for children who had received the Abecedarian Approach in the first five years of life. This three-part cognitive/social/motivational model makes intuitive sense and relates closely to the other observations in this article. The fact that these three predictors explain, respectively, only 77.94% and 38.15% of the preschool effect on education at age 21 for the Perry Preschool project and the Chicago Child-Parent Centers [43] (p. 432) suggests that the Abecedarian intervention is more tightly aligned and its program more congruent to the cognitive/social/motivational hypothesis. This analysis does not comment on the positive effects of programs during the school years, rather, it shows that among children randomly assigned to receive or not receive the Abecedarian Approach in the early years, the cognitive/social/motivational predictive model is very accurate in forecasting years of education at age 21.

### 3.16. Abecedarian Training and Caregiver Behavior

The Abecedarian Approach, like many interventions, has a logic map that assumes that the educational program first is incorporated into an adult’s behavior and reaches the target child as the adult and child interact. In many studies the child’s behaviors are measured but not the adult’s. However, one randomized Abecedarian study in the USA measured the behavior of child care providers who were trained in the Abecedarian Approach and implemented it in family child care homes. The training of caregivers had statistically significant impacts on caregiver behaviors. That is, compared with the control group, the Abecedarian trained caregivers had substantially higher frequencies of rich oral language interactions, interactions to support children’s understanding of vocabulary or concepts, and responsiveness to the children [44]. None of the provider baseline characteristics significantly predicted the three provider outcomes. The fact that Abecedarian training makes a significant difference in how adults talk to and interact with the children in their care confirms that the logic pathway, training > adult behavior > child developmental progress, is functioning as desired. It is within the adult-child relationship that positive child development occurs and thrives.

In a Canadian qualitative study of caregivers and parents, the main themes that emerged were strengthened relationships between parents and program staff, as well as between parents themselves; increased awareness among parents about early development and of their role in supporting child development; and opportunities for parents’ personal growth [8]. The findings suggest that high quality early child intervention programs, such as the Abecedarian Approach, can positively impact all the adults who interact with the children.

## 4. Conclusions

Multiple Abecedarian studies [2,45] have shown that early and broad intervention early in life for at-risk children yields early and long-lasting positive outcomes. Among these are the social and mental health benefits that prepare the child for school and life success.

If an academically at-risk child has daily had individualized games, reading sessions, and information-filled caregiving/language interactions from responsive adults during three to five years of educational child care and/or home life, then the experience of school (even though it is taught mostly in groups and is not individualized) is likely to be easy to comprehend and respond to. Successfully playing a game with a caregiving adult will map onto the new experience of doing a lesson with a teacher. From his or her early experience, the child expects to receive adult input, to pay attention, to respond, and to succeed. This is the social, attitudinal, and learning dispositions advantage the child brings to school. Moreover, the child enters school developmentally on track (from mastering all the knowledge and skills incorporated in the Interaction Games and other activities) and does not have to play catch-up. This is the cognitive advantage that enables the child to benefit from each succeeding experience as it presents itself.

## Figures and Tables

**Table 1:** Abecedarian input-output map.